# Concurrent TMS–fMRI reveals dynamic interhemispheric influences of the right parietal cortex during exogenously cued visuospatial attention

**DOI:** 10.1111/j.1460-9568.2010.07580.x

**Published:** 2011-03

**Authors:** Klaartje Heinen, Christian C Ruff, Otto Bjoertomt, Bertram Schenkluhn, Sven Bestmann, Felix Blankenburg, Jon Driver, Christopher D Chambers

**Affiliations:** 1 UCL Institute of Cognitive Neuroscience and Wellcome Trust Centre for Neuroimaging, University College London17 Queen Square, London WC1N 3AR, UK; 2 Laboratory for Social and Neural Systems Research, University of ZurichIEW, Zurich, Switzerland; 3 Sobell Department for Motor Neuroscience and Movement Disorders, Institute of Neurology, University College LondonLondon, UK; 4 School of Psychology, Cardiff UniversityWales, UK; 5 Department of Neurology and Bernstein Center for Computational NeuroscienceCharité, Berlin, Germany

**Keywords:** angular gyrus, functional magnetic resonance imaging, humans, reorienting, transcranial magnetic stimulation, visual attention

## Abstract

We used concurrent transcranial magnetic stimulation and functional MRI (TMS-fMRI) during a visuospatial cueing paradigm in humans, to study the causal role of the right angular gyrus (AG) as a source of attentional control. Our findings show that TMS over the right AG (high vs. low intensity) modulates neural responses interhemispherically, in a manner that varies dynamically with the current attentional condition. The behavioural impact of such TMS depended not only on the target hemifield but also on exogenous cue validity, facilitating spatial reorienting to invalidly cued right visual targets. On a neural level, right AG TMS had corresponding interhemispheric effects in the left AG and left retinotopic cortex, including area V1. We conclude that the direction of covert visuospatial attention can involve dynamic interplay between the right AG and remote interconnected regions of the opposite left hemisphere, whereas our findings also suggest that the right AG can influence responses in the retinotopic visual cortex.

## Introduction

The direction of covert visuospatial attention can modulate visual cortex processing, putatively under the control of higher-level control structures such as the parietal cortex. A classic method of manipulating covert visuospatial attention is via salient cues that may correspond to the location of a subsequent visual target (on ‘validly’ cued trials), or which trigger a shift of attention between cue and target locations on ‘invalidly’ cued trials (e.g. Posner *et al.*, 1980). Functional neuroimaging and neuropsychological studies have implicated an ‘inferior’ or ‘ventral’ attention network [including the angular gyrus (AG) and/or temporal parietal junction particularly in the right hemisphere] during shifts of attention on invalidly cued trials (see Friedrich *et al.*, 1998; Corbetta *et al.*, 2000; Macaluso *et al.*, 2002; Thiel *et al.*, 2004; Kincade *et al.*, 2005; Mevorach *et al.*, 2006; Vuilleumier *et al.*, 2008). It has thus been suggested that the right AG (and temporal parietal junction) areas may be crucial for controlling attentional reorienting (or for acting as part of a ‘circuit breaker’) (Corbetta & Shulman, 2002), potentially via modulation of remote but interconnected visual cortex (Desimone & Duncan, 1995; Kastner *et al.*, 1998).

Functional neuroimaging alone cannot establish a causal role for specific brain regions, as techniques such as functional magnetic resonance imaging (fMRI) record rather than manipulate neural function. In contrast, transcranial magnetic stimulation (TMS) offers a non-invasive means of targeting a particular brain region with a causal intervention and then examining the behavioural consequences (e.g. Pascual-Leone *et al.*, 2000; Chambers & Mattingley, 2005). Recent TMS studies of visual attention implicated the right AG as a crucial node for directing attention, as applying TMS over this site can reliably alter the behavioural consequences of invalid spatial cueing (Rushworth *et al.*, 2001; Chambers *et al.*, 2004; Chambers & Mattingley, 2005).

However, purely behavioural TMS studies cannot, in isolation, determine whether the impact of TMS applied to a targeted local site reflects purely local changes in activity at that site alone. TMS effects might also involve causal influences of the targeted area upon interconnected regions (e.g. see Driver *et al.*, 2009). Indeed, in the case of spatial attention, it is plausible that such extended networks may include homologous regions in the opposite hemisphere (e.g. see Kinsbourne, 1977).

**FIG. 1:**
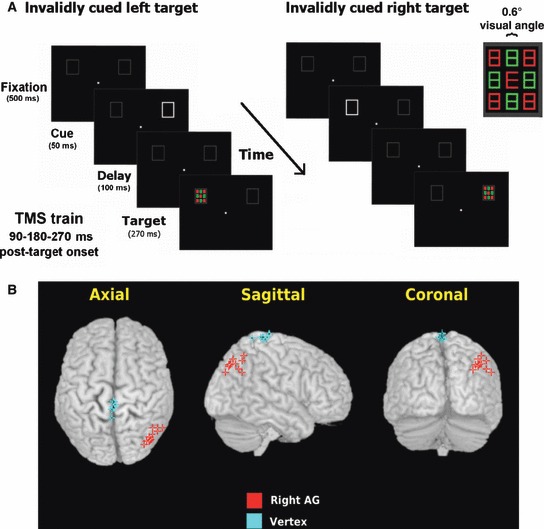
Experimental task and TMS sites. (A) Schematic sequence of visual displays in the spatial cueing paradigm, shown here for invalidly cued left or right targets. Each trial commenced with a central fixation point and two placeholders in upper quadrants at 11° eccentricity. A brief (50 ms) exogenous cue then appeared randomly in the left or right hemifield, as a brightening of the placeholder (to 100% contrast) on one side. Participants were instructed simply to ignore this cue, which did not predict the target hemifield (i.e. validly and invalidly cued targets were equiprobable, 50% each). Following a brief (100 ms) delay, the target was presented within one of the placeholders, selected randomly. Invalidly cued trials (as displayed here) were of particular interest, as they require reorienting of attention between the cue in one hemifield and the target subsequently appearing on the opposite side. Each target (see large example shown separately in expanded inset at top-right of figure) comprised a red/green 3 × 3 grid (1.95° × 2.75°), with a central letter (A, E, F or P; 0.64° × 0.92°) composed of sub-elements from the flanking digits. On each trial, a triple burst of TMS was applied at 90–180–270 ms after target onset, at low or high intensity (see main text). (B) TMS sites were identified *a priori* in each participant based on neuroanatomical landmarks (see text for details). Here Montreal Neurological Institute-converted coordinates for the right AG (red) and vertex (blue) sites per participant are projected onto a normalized standard brain (see Supporting Information Fig. S1 for TMS site specification within each participant in their native space, with the intraparietal sulcus marked in each individual). For interpretation of references to color in the figure legend, please refer to the Web version of this article.

Recent advances now allow concurrent combination of TMS with fMRI (Bohning *et al.*, 1999; Baudewig *et al.*, 2001; Sack *et al.*, 2007; Bestmann *et al.*, 2008b; Driver *et al.*, 2009). By applying TMS to a local site during whole-brain fMRI, this approach can potentially reveal any remote consequences of TMS on neural activity, as indexed by blood-oxygen-level dependence (BOLD) signals, and how these might relate to behavioural change. Here we applied this approach to study the impact of right AG stimulation on behaviour and brain activity in an event-related visuospatial attention paradigm (Fig. 1). We were especially interested in any potential interhemispheric effects of the TMS upon BOLD signals (Kinsbourne, 1977; Bestmann *et al.*, 2008b; Blankenburg *et al.*, 2008), and also in any influences of parietal stimulation upon the early visual cortex that may relate to TMS-induced changes in visual performance.

To establish time-locked effects of cortical stimulation, we compared high- vs. low-intensity TMS over the right AG during selective spatial attention in an event-related design. Note that comparing TMS intensities is better controlled than merely comparing AG TMS vs. none. Moreover, as a further control we also applied high- vs. low-intensity TMS to a control site (vertex) that should not produce any specific effects, in a separate experiment.

## Materials and methods

### Participants

Twelve healthy participants (age 24–36 years; five males, all right-handed, normal or corrected vision) took part in the behavioural (TMS only) part of the study. Five of this group (four males) also volunteered to undertake the equivalent procedure in the scanner during concurrent TMS–fMRI sessions, plus individual retinotopic mapping (see below). All provided informed consent to participate, following screening for medical contraindications to MRI and TMS. The study was approved by the local ethics committee (National Hospital for Neurology and Neurosurgery & Institute of Neurology Joint Research Ethics Committee, London, UK) and was conducted to conform with the Declaration of Helsinki.

### Stimuli and procedure

Participants undertook a psychophysically-calibrated spatial cueing task (see Fig. 1A) in which the accuracy of (unspeeded) perceptual discrimination was the primary dependent variable. Six equiprobable conditions were generated through the factorial combination of exogenous spatial cue type (valid or invalid), target hemifield (left or right) and TMS intensity (high or low). Separate experiments were conducted with TMS applied over the right AG or a control site (vertex). The use of a vertex control site that should not be expected to induce any specific effects has become standard in many TMS studies (e.g. Harris *et al.*, 2008; Muggleton *et al.*, 2008; Kalla *et al.*, 2009), including for recent concurrent TMS–fMRI work (e.g. Ruff *et al.*, 2006, 2008, 2009).

Each trial in the main experiment commenced with onset of a central fixation point and two lateral placeholders for 500 ms (11° eccentricity, one in each hemifield, see Fig. 1A). A brief exogenous peripheral cue (50 ms) then appeared randomly in the left or right hemifield as a lateral brightening of one placeholder (83% increase in contrast). Participants were instructed to ignore this exogenous cue, which did not predict the target hemifield. Following a short delay period (100 ms), the target ensemble was presented within one of the placeholders, randomly selected on left or right in an event-related manner. The target array (1.95° × 2.75°) comprised a target letter (A, E, F or P; 0.64° × 0.92°) coloured red or green, flanked by a surrounding grid of red and green ‘8’-shapes with alternating red/green colours (see expanded inset at top right of Fig. 1A). The purpose of these surrounding flankers and the task was to ensure that the target discrimination required focused spatial attention, and that the target ensemble should drive a substantial response in the early visual cortex including retinotopic areas (see Noesselt *et al.*, 2002).

On each trial, participants identified the target letter within the centre of the target array that appeared on the left or right, as accurately as possible within a 3-s response interval, by pressing one of four keys on either a standard keyboard (outside the scanner) or an magnetic resonance-compatible response box (inside the scanner). The colour of the target letter was randomly red or green, with this colour also selected for the surrounding elements on the outside corners of the target ensemble (see expanded insert at top right in Fig. 1A). The target ensemble flickered (with 50 ms on and 40 ms off periods), which was again intended to enhance the stimulus-evoked response in the early visual cortex.

In each experiment (AG or vertex TMS), participants undertook two to three sessions of TMS outside the scanner (a total of six to nine blocks of 128 trials each) and one session of concurrent TMS–fMRI (three blocks of 128 trials). Each block included 16 trials per condition in the three-way factorial 2 × 2 × 2 design, i.e. crossing TMS intensity (high, low) with cue validity (invalid, valid) and target hemifield (left, right). For each TMS site, each participant thus completed 96–144 trials per condition outside the scanner, and subsequently 48 trials per condition inside the scanner for those participants who also underwent concurrent TMS–fMRI.

Participants were trained on the task in an initial practice session with feedback on response accuracy in the four-choice letter discrimination task. In a subsequent calibration session, the luminance of the elements surrounding the target letter (see Fig. 1A) was calibrated psychophysically in each participant to yield approximately 70% correct identifications on trials without cues (resulting in surround luminances of 15–20% of target letter luminance, depending on participant performance during calibration).

Individual TMS resting motor thresholds were obtained in a separate session, via stimulation over the right M1 ‘hotspot’ for inducing a visible twitch of the first dorsal interosseus (mean resting motor threshold with conventional TMS coil 56 ± 10% of maximal stimulator output; or with the non-ferrous MR-compatible coil that had a longer cable 67 ± 10% of maximal output).

On each trial, a burst of three TMS pulses was applied at 11 Hz, with single pulses at 90, 180 and 270 ms after target onset. Note that TMS was applied shortly after target onset here because validly and invalidly cued trials only differ once the target appears at the cued or uncued location. Previous purely behavioural TMS work has established a role of the right AG during this time window for covert attentional reorienting (Rushworth *et al.*, 2001; Chambers *et al.*, 2004).

The TMS was delivered equiprobably at either a low (40% motor threshold, which should be neurally ineffective, but provided a control for any non-specific aspects associated with TMS delivery and expectation of such delivery) or a high (120% motor threshold, expected to be neurally effective) intensity. These TMS intensities were randomly intermingled in an event-related design, and administered over the AG, or in a separate experiment to the vertex control site (see Fig. 1B; see also Supporting Information Fig. S1 for more detail of whether the stimulation sites fell within native space for each individual participant). In total, participants received 1152 TMS pulses during each fMRI session, of which 50% were low intensity and hence expected to be neurally ineffective. This total accords with published TMS safety guidelines (Rossi *et al.*, 2009). The intertrial interval was designed with scanning constraints in mind, varying randomly between three and four scanning repetition times (TRs) (see below) and thus corresponding to 7.3–9.7 s. During the intertrial interval, only the grey central fixation square and peripheral placeholders were visible. A TMS–fMRI session consisted of three runs, each lasting approximately 18 min. Between these runs, the participant rested for 3–5 min. Retinotopic mapping sessions, acquired separately, took approximately 20 min.

### Localisation of TMS stimulation sites

Scalp coordinates for the stimulation sites were first located outside the scanner via the Brainsight Frameless stereotaxic system and software package (Rogue Research, Montreal, Canada), using the native space of each participant's own T1-weighted anatomical MR image. The right AG was identified in each participant (and then marked on their scalp) as the dorsolateral termination of the superior temporal sulcus, which bifurcates the AG in the inferior parietal lobule. See red symbols in Fig. 1B for an overview of the TMS sites in normalized space, plus Supporting Information Fig. S1 for site specification in individual native space for each subject, with the intraparietal sulcus also marked. The selected AG site corresponded to mean Montreal Neurological Institute coordinates of 40, −73, 44 (SE = 5.3, 3.6, 3.4). The control ‘vertex’ site was defined as the medial junction of the bilateral central sulci and the longitudinal fissure (mean Montreal Neurological Institute = 0, −34, 78; SE = 0.9, 8.5, 2.8; see blue symbols in main Fig. 1B and Supporting Information Fig. S1).

### Functional magnetic resonance imaging

During scanning, the visual display was backprojected onto a screen viewed via a mirror mounted on top of the MR head coil. Functional images were acquired on a 1.5 T MR system (Siemens Sonata, Erlangen, Germany), with a single channel receive head array. T2*-weighted echo planar image (EPI) volumes were acquired every 2.43 s covering the whole brain down to the cerebellum (27 transversal slices; α = 90°; repetition time (TR), 90 ms; echo time (TE), 42 ms; 64 × 96 matrix; voxel size 3.00 × 3.00 × 3.75 mm; 2.5 mm slice thickness plus a slice distance of 50%). All volumes were oversampled by 50% in the phase-encoding direction. This increased the field of view, allowing the Nyquist ghosting associated with the physical presence of the MR-compatible TMS coil to be relocated outside the brain image (see also Ruff *et al.*, 2006, 2008, 2009; Bestmann *et al.*, 2008a; Blankenburg *et al.*, 2008). The first five volumes were discarded to allow for T1 equilibration effects.

Data for our retinotopic analysis (see below) were acquired in the same scanner. For this we used a custom-built visual surface coil as a receiver (Nova Medical Inc., Boston, MA, USA) to enhance the signal-to-noise ratio over the occipital cortices. The use of an occipital surface MR coil is well established and has become standard for retinotopic mapping protocols, with the aim of increasing signal-to-noise in the visual cortex. This was particularly appropriate for the current experiment, as time constraints due to the many other aspects of our study required us to employ a relatively brief meridian-mapping retinotopic protocol (see below). The standard Siemens body coil was still used for transmission. T2*-weighted EPI volumes were acquired every 2.52 s and covered 27 transverse slices (α = 90°; repetition time (TR), 90 ms; echo time (TE), 50 ms; 64 × 64 matrix; voxel size 3 × 3 × 3 mm; 2 mm slice thickness including a slice distance of 50%). At the end of the scanning session, whole-brain EPIs were acquired with the body coil using the same orientation, to facilitate spatial co-registration of the spatially-restricted image series. Although our use of different MR coils for the retinotopic mapping aspect vs. the main fMRI experiment (as also implemented in Ruff *et al.*, 2006, 2008; Liu *et al.*, 2005) may limit co-registration to some extent, this should be offset by the greatly enhanced signal-to-noise ratio for retinotopic regions when using the occipital surface coil for retinotopic mapping, during which only occipital areas were of interest.

Whole-head T1-weighted anatomical images were acquired after the experiment using an eight-channel phase array coil and a 3D modified driven equilibrium fourier transform (MDEFT) sequence with an isotropic resolution of 1 mm^3^ (Deichmann *et al.*, 2004).

### Interleaved TMS–fMRI

Following localization of the TMS site on the participant's scalp outside the scanner using Brainsight neuronavigation (see above), the participant was placed in the scanner and the TMS coil carefully fixed against the marked site on the head using a custom coil holder. The TMS coil was positioned over the marked location with the handle oriented 45° from the vertical midline in a posterio-medial direction. Foam-padded cushions were used to restrict head movements. Test TMS pulses (single pulses and triple bursts like those used in the experiment) assessed any potential problems with peripheral nerve stimulation or induced motor twitches; none was elicited.

A Super Rapid stimulator (Magstim, Whitland, UK) was used to generate TMS, together with an MR-compatible, non-ferrous figure-of-eight coil with a wing diameter of 70 mm. The Magstim stimulator was located in a Faraday cage and connected to the TMS coil through a custom filter box (Magstim, Dyfed, UK) and a cable fitted with ferrite sleeves (Wuerth Elektronik, Waldenburg, Germany). The TMS coil was connected to the stimulator in parallel with a high-voltage relay (ES 9486; The Magstim Company). During EPI acquisition, the relay was closed, thereby preventing any residual leakage in current flow from the stimulator (see Weiskopf *et al.*, 2009). The relay was opened at 270 ms prior to TMS discharge and closed again immediately after termination of the last TMS pulse of a trial.

The TMS was applied in a burst of three pulses (11 Hz), starting at 90 ms following target onset. Each pulse train was synchronized with EPI acquisition by time-locking the target array onset with the acquisition of the slice preceding TMS. Each perturbed slice during TMS was subsequently removed and replaced by temporal interpolation of the signal values for the same slices in the preceding and subsequent volume (see analysis) equally often throughout a scanning session, to avoid any systematic errors in slice-to-slice variance. The TMS pulses were temporally separated from any slice selection gradients or excitation pulses and EPI readout gradients (see Bestmann *et al.*, 2008a, for details).

Throughout scanning, eye position, blinks and pupil diameter were monitored with an ASL 504 Remote Infrared Eyetracker (Applied Science Laboratories, Bedford, USA) (see also Ruff *et al.*, 2006, 2008) at 60 Hz via long-range optics. Eye tracking was similarly undertaken for all sessions outside the scanner. Online inspection confirmed adherence to central fixation, as expected in these highly practiced (but naive) participants. Within the scanner, a semi-translucent screen was employed for back projection of visual stimuli. This screen was viewed via a mirror and spanned approximately 30° × 22° of visual angle. Participants wore earplugs to reduce acoustic noise from the scanner and the auditory TMS ‘click’ when discharged. All visual stimulation, TMS triggering, intensity regulation and relay settings were controlled using the Cogent toolbox (http://www.vislab.ucl.ac.uk/cogent_2000.php) within Matlab (Mathworks, MA, USA).

### Data analysis

#### Behaviour

Behavioural data were analysed separately for each TMS site (AG, vertex), initially including all sessions inside and outside the scanner, but for completeness also separately when considering only those sessions undertaken by participants inside the scanner. A three-way anova was applied in accord with our balanced factorial design, with factors of TMS intensity (high, low), cue validity (invalid, valid) and target hemifield (left, right). All eight resulting conditions were equiprobable. The behavioural performance of one (non-scanned) participant was excluded as an outlier (> 4 SD from the mean). Our findings (see below) confirmed that the behavioural effects of TMS replicated from outside to within the scanner.

#### Functional MRI data

The analysis of imaging data was undertaken using SPM5 (http://www.fil.ion.ucl.ac.uk/spm). The fMRI data were corrected for any possible TMS artefacts by first identifying and replacing slices acquired during TMS with temporal interpolation of the signal values for the same voxels in the preceding and subsequent volume (see also Ruff *et al.*, 2006, 2009; Bestmann *et al.*, 2008a, b). In addition, any remaining slices ≥ 2 SDs from the session mean were replaced in the same way (typically < 1%).

The first five volumes of each fMRI run were discarded. All subsequent volumes were realigned to the sixth volume to correct for interscan movement, and were spatially normalised to Montreal Neurological Institute anatomical standard space. The normalised images were spatially smoothed for the whole-brain analysis (8 mm full width at half-maximum Gaussian kernel) in accord with the standard SPM approach. Low-frequency fluctuations were removed from the analysis using a temporal high-pass filter (64 s cut-off) and images were normalised for global intensity. The normalization and smoothing preprocessing steps were omitted for individual retinotopic analyses (see below), as is standard.

The preprocessed images were then fed into a General Linear Model. For each session, data were analysed with a separate regressor for each of the eight experimental conditions in the 2 × 2 × 2 factorial event-related design. Blink onsets were extracted from the eye-tracking data, after being identified using a band-pass filter on pupil-reflex data (80–1000 ms, 100% signal loss of pupil and corneal reflection). Subsequently, the blink onsets were entered as regressors of no interest to account for any blink-related variance in the fMRI data and thereby ensure that this could not contribute to the effects of the experimental conditions on BOLD responses (see also Ruff *et al.*, 2006, 2008, 2009). BOLD responses were modelled with the canonical haemodynamic response function in SPM5 and its temporal derivative, by convolution of the regressors. Other regressors of no interest included mean pupil size per scan [delayed by 2 repetition times (TRs)] and head movement parameters (six regressors). Thus, any variance in the fMRI data attributable to blinking, pupil size or any head motion shared with the independent variables could not contribute to the subsequent contrasts of interest, being regressed out separately.

The height threshold for SPM images was initially set at T > 3.09 uncorrected, but importantly all whole-brain reported effects passed the threshold of *P* < 0.05 corrected for multiple comparisons at cluster level across the whole brain. For closer analysis of BOLD data in the vicinity of the right AG TMS coil, a small region of interest (ROI) volume was inspected (now with false discovery rate correction for its extent, at *P* < 0.05 corrected), comprising a sphere of 10 mm radius centred around the mean Montreal Neurological Institute coordinates of the stimulated right AG (40, −73, 44).

### Retinotopic meridian mapping

Retinotopic meridian mapping was undertaken in an additional fMRI session using the occipital surface MR coil. We used a standard brief retinotopic mapping protocol, in which horizontal and vertical meridian checkerboards were used to functionally delineate areas V1–V3 (Kastner *et al.*, 1998) for each of the individual scanned participants using SurfRelax. We did not use more extended retinotopic mapping here (such as polar-angle mapping or extension to higher retinotopic regions such as V7), as our participants already had to undergo a prolonged TMS and TMS–fMRI protocol, so that time constraints dictated the quicker meridian-mapping approach. It might be interesting to extend the present approach to further retinotopic areas in future, with more extensive retinotopic mapping protocols. For now, the relatively brief meridian-mapping protocol was adequate to distinguish the borders of V1, V2 and V3, both dorsally and ventrally, in each scanned participant. These early retinotopic areas were of interest given our question of whether AG TMS might affect the early retinotopic regions that responded to our visual targets, with any such remote effects of parietal TMS on the early visual cortex potentially varying with attentional condition as we tested.

The mean percentage signal change in each condition from the main experiment was then extracted, using MarsBar (http://marsbar.sourceforge.net/), specifically for the target-responsive voxels within ventral V1–V3 in the left and right hemispheres, for right or left target conditions, respectively. Voxels responding to our left or right target stimuli, within retinotopic areas, were defined by masking the retinotopic maps with intrinsic contrasts of left vs. right targets from the main experiment. Note that these left/right target contrasts used to define contralateral target responses within (separately mapped) retinotopic areas V1–V3 were fully independent of the contrasts subsequently used to inspect any TMS and/or validlity affects in the retinotopic cortex, i.e. we looked at TMS and/or validity effects separately for the left and right retinotopic cortex (in the presence of their contralateral target), thereby avoiding circularity for our critical tests (cf. Kriegeskorte *et al.*, 2009, 2010).

As a control to test for the specificity of any TMS and/or validity effects within target-responsive ventral V1–V3, we also examined the response of dorsal V1–V3, to which our upper-quadrant visual stimuli (see Fig. 1A) should not project.

## Results

### Behaviour

**FIG. 2:**
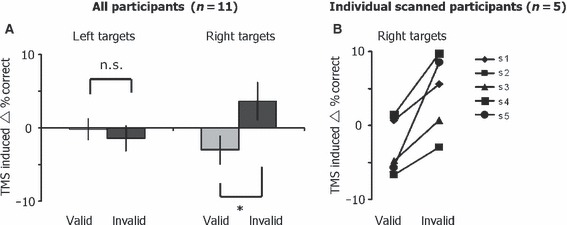
Behavioural effects of right AG TMS (high- vs. low-intensity) depended on target side and cue validity. *, *P* < 0.01. (A) For right targets (rightmost pair of bars), high- vs. low-intensity TMS of the right AG enhanced the identification of invalidly cued right targets (dark bar at far right) relative to validly cued right targets (adjacent light bar) (*F*_1,10_ = 17.36 *P* = 0.002). This pattern was observed in 10 out of 11 participants, and in all five of the subsequently scanned participants. In contrast, TMS did not affect the identification of the left targets (leftmost bars), either for validly or invalidly cued conditions (see leftmost pair of bars). Data shown here are pooled across outside-scanner and inside-scanner situations, but there were no significant differences between these. By contrast, vertex TMS in the control experiment had no significant impact on performance (data not shown for brevity, see main text). (B) The enhanced identification of right targets on invalid trials during TMS of the right AG was consistent across all participants subsequently scanned, as shown here for their data pooled across being inside or outside the scanner. There were no significant differences in behavioural outcome between these two contexts.

Behaviourally, we compared visual discrimination accuracy for targets on validly vs. invalidly cued trials in 11 participants (five of whom also volunteered for the scanning study) for each hemifield. As expected, performance was reliably reduced on trials with invalid cues [63% correct vs. 77% with valid cues, *t*(10) = 7.4, *P* < 0.001], as apparent in all subjects. We next considered any impact of right AG TMS (high vs. low intensity) on the accuracy of visual discrimination for left or right targets in the different cueing conditions. For right targets, a significant interaction between TMS intensity and invalid/valid cueing was observed (*F*_1,10_ = 17.4, *P* < 0.005). Figure 2A plots the changes in accuracy induced by right AG TMS (i.e. high- minus low-intensity differences in accuracy). For the right targets (rightmost two bars in main Fig. 2A), the dependency of TMS effects on precueing is evident as an improvement in accuracy for right invalidly- cued compared with validly-cued targets. There was no such behavioural impact of right AG TMS for left targets (see leftmost two bars in Fig. 2A), yielding a significant three-way interaction between TMS intensity, validity and target hemifield (*F*_1,10_ = 4.9, *P* < 0.05) for behavioural accuracy.

This effect of AG stimulation on validity effects for right targets was consistent across the sample, being observed in 10 out of 11 participants. The five participants who subsequently took part in our concurrent TMS–fMRI experiments (which included individual retinotopic mapping) also exhibited this behavioural TMS effect consistently, for all five outside the scanner and for all except one within the scanner. The latter subject, P4, still did show the key pattern when their behavioural data from inside and outside the scanner were pooled. See Fig. 2B for individual behavioural data from the five scanned participants.

Thus, the impact of right AG TMS upon behaviour was consistent across participants. The details of this behavioural impact differ in some respects from previous work on effects of parietal TMS, probably due to differences in the exact spatial cueing paradigms and TMS protocols (cf. Rushworth *et al.*, 2001; Chambers *et al.*, 2004; Chambers & Mattingley, 2005). However, the behavioural effects within the current paradigm were significant and replicable and, as will be shown, they related to the fMRI pattern found below. The most striking impact was a specific improvement in accuracy for right invalidly cued targets, which indicates enhanced rightward reorienting on such trials, due to the right AG TMS.

No behavioural impact of TMS was observed in the control experiment, where TMS was applied to the vertex in the same participants (*P* > 0.8 for the three-way interaction of target hemifield × TMS intensity × cue validity; *P* > 0.35 for the two-way interactions of TMS intensity × cue validity, for either target hemifield considered separately).

To summarize the behavioural outcome, high- vs. low-intensity TMS over the right AG facilitated rightward reorienting of attention, leading to improved accuracy for right invalidly cued targets in particular. This effect (which led to a three-way interaction) was observed both outside and inside the scanner and was consistent across participants, while being absent during vertex TMS.

### Functional imaging results during TMS

By employing concurrent TMS–fMRI, we were able to measure BOLD responses during TMS of the right AG in the same paradigm as above. Recall that our behavioural analysis already showed that TMS over the right AG specifically affected performance for right targets in a manner that depended on cue validity (a performance benefit specifically for right invalidly-cued targets, leading to the three-way interaction). Our whole-brain SPM analysis therefore also considered the three factors of TMS intensity (high vs. low), cue validity (invalid or valid) and target hemifield (right or left), and tested initially for the analogous three-way interaction pattern as found behaviourally.

For this initial whole-brain SPM analysis, we focus on results that reached cluster-corrected significance (less prominent patterns are detailed in full within Supporting Information Table S1). For completeness, we further assessed a spherical ROI located beneath the TMS probe over the right AG. Finally, after the SPM whole-brain analysis, we then move on to analyse the retinotopic visual cortex as functionally defined in individuals, specifically for those voxels representing the visual locations of the target stimuli, to determine if AG TMS could impact the response of the early visual cortex to our targets, in a condition-dependent way that might also mirror the interaction found behaviourally above.

#### Context-dependent interhemispheric effects of right angular gyrus stimulation on the BOLD response in the left hemisphere

**FIG. 3:**
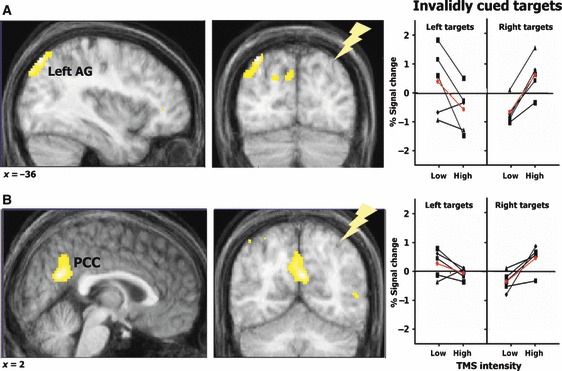
Effects of right AG TMS on the BOLD response depend on the trajectory of spatial attention. (A) The three-way interaction SPM contrast, testing for an impact of high minus low TMS intensity that was larger for invalidly cued right targets in particular (analogous to the behavioural pattern), revealed such an interaction at cluster-corrected whole-brain signficance for the left AG (peak at *x* = −36, *y* = −76, *z* = 50) and (B) in the bilateral posterior cingulate cortex (peak at *x* = 2, *y* = −60, *z* = 25) extending to the left precuneus (*x* = −8, *y* = −74, *z* = 33). This TMS-induced response (i.e. difference between high minus low TMS intensity conditions) was not observed during stimulation of the control site (vertex). As plotted in the line graphs on the right, to illustrate the form of the interaction (see main text), the percentage signal changes of interest from the right AG TMS–fMRI experiment (shown for invalidly cued trials) extracted from the peak for (a) left AG or (b) left PCC, reveal a TMS-induced increase in BOLD response for invalidly cued right targets, combined with a TMS-induced reduction for trials with left targets. This pattern was consistent across participants. Furthermore, the effects were selective for invalidly cued trials, with a less pronounced opposite trend observed for validly cued targets (not shown for brevity). These results reveal right AG-TMS-induced modulation of activity in the left AG (and posterior cingulate/left precuneus) that depended on the validity of the cue as well as target side, and was contingent on the direction of spatial reorienting, showing a similar three-way interaction to that found for the behavioural impact of right AG TMS.

Analogous to the pattern that we found in behaviour, we specifically tested for regions where the TMS-induced (high > low) impact on BOLD was more pronounced for invalid than valid trials, and more so for right than left targets. This whole-brain, three-way interaction contrast revealed a cluster in the left AG, contralateral to and strikingly symmetric with the site of TMS (see Fig. 3A); plus in the bilateral posterior cingulate cortex (Fig. 3B) extending to the left precuneus (Fig. 3A, coronal slice; see Supporting Information Table S1).

These clusters in the left AG and posterior cingulate cortex passed whole-brain correction at the cluster level (see Supporting Information Table S1) for the same three-way interaction contrast that had arisen in behaviour. In order to illustrate the critical aspect of the pattern for this high-level interaction in the BOLD data from these regions (see Kriegeskorte *et al.*, 2010), without altering the *P*-values already yielded by whole-brain cluster correction (thereby avoiding circularity), we show the extracted percent signal change for left or right invalidly cued targets as a function of TMS intensity (low or high) in the plots on the right in Fig. 3 for each subject. For simplicity, we focus on data for the invalidly cued trials that were of main interest, as those were the trials designed to induce reorienting of attention. The line graphs in Fig. 3 illustrate that high TMS increased the BOLD signal for right invalid cued targets in all five participants, whereas no such increase (but even a decrease instead) was observed for left invalid cued targets. There was no such pattern for the valid trials. Therefore, the high-level interaction involved a TMS-dependent increase in BOLD specifically for trials with right invalidly cued targets. No such differential activations were observed in the vertex-TMS control experiment (e.g. for the same left AG cluster, *P* > 0.5 for the same three-way interaction contrast). With right AG TMS, a similar pattern was also observed for the cingulate cortex/left precuneus cluster (Fig. 3B) as for the left AG (Fig. 3A), which was again absent during vertex-TMS (*P* > 0.6, n.s.).

For completeness, we also examined activity immediately beneath the TMS probe in the right AG. When applying a small volume correction for a sphere of 10 mm diameter centred on the mean coordinate of AG stimulation (40, −73, 44), the highest-level three-way interaction yielded no significant voxel clusters. However, a two-way interaction contrast for right targets only (TMS intensity × validity) did reveal a significant cluster (*P* = 0.038 with false discovery rate correction). Examination of the parameter estimates from the peak of this cluster (at *x* = 40, *y* = −72, *z* = 45) revealed that, in the vicinity of the site of right AG stimulation, high minus low TMS enhanced the BOLD response for invalidly cued right targets, again reminiscent of the behavioural facilitation in this specific condition. We note that concurrent TMS–fMRI can lead to less MR sensitivity immediately under the TMS coil (as for the right AG here) due to technical reasons (see Bestmann *et al.*, 2003). That technical aspect alone might explain why the full three-way interaction pattern seen for the left AG (Fig. 3A) did not reach full significance for the right AG here, only the related two-way interaction of TMS × validity for right targets considered alone.

#### Retinotopic analysis of condition-dependent TMS impacts upon visual areas

If the right AG is a source of attentional control that modulates visual processing during spatial attention (as suggested by our behavioural results here, see above), then TMS of this region may affect activity in the retinotopic visual cortex proper. To obtain a more detailed view of any remote effects of right AG TMS upon the retinotopic visual cortex in particular, as a function of attentional condition, we next examined the BOLD percent signal changes for individually defined areas V1–V3, specifically for those voxels within these mapped areas that responded contralaterally to our upper quadrant peripheral visual stimuli.

Meridian mapping (see Materials and methods) was used to define borders between successive retinotopic areas V1–V3, independently of the main experiment. Intrinsic stimulus localizers (the main effects of left vs. right targets) identified those voxels within each of these retinotopic areas that responded to contralateral targets, thus producing individual ROIs (retinotopic ROIs) that represented the location of our upper quadrant visual stimuli for ventral V1, V2 and V3. Data were then extracted from each individual retinotopic ROI for the main experiment, for statistical analyses outside SPM, now considering left and right target trials separately (for right or left target-responsive retinotopic ROIs, respectively), in order to determine any TMS and/or validity effects in these ROIs responding to contralateral targets. Thus, we now tested the ROIs for contrasts that are fully independent of those that defined them.

**FIG. 4:**
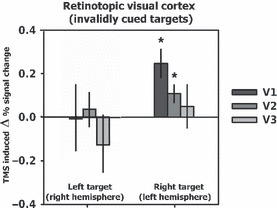
Effects of right AG TMS on retinotopic activity for invalidly cued targets. TMS-induced changes (high minus low intensity difference) in the BOLD response for target-responsive voxels in retinotopically mapped ventral V1–V3, for invalidly cued targets in either hemifield (i.e. for left targets in the right ventral visual cortex or for right targets in the left ventral visual cortex). Recall that, behaviourally, high-intensity stimulation of the right AG facilitated reorienting to right invalidly cued targets, as assessed by perceptual discrimination accuracy. In the same condition (i.e. right invalidly cued targets), such targets were associated with a TMS-induced increase in BOLD response for the contralateral visual cortex, in left V1 and V2 where the right targets are represented (see bars towards right of the figure; asterisks mark significant effects). There was no such effect in left V3 or in right V1–V3 for left invalidly cued targets (see three bars on left of the figure). Analogously to the behavioural pattern, the impact of right AG TMS on the visual cortex was thus specific to right but not left invalidly cued visual targets, and correspondingly affected the left visual cortex in particular. See also main text for confirmation that these TMS-induced effects on the left visual cortex were not found for validly cued targets, or within control regions of dorsal V1–V3 that do not represent the upper-quadrant target locations retinotopically.

These retinotopic ROI analyses revealed condition-dependent effects of (high minus low) right AG TMS on BOLD responses to invalidly cued right targets in the left retinotopic visual cortex (see percent changes in BOLD signal shown in rightmost three bars in Fig. 4). Specifically, high-intensity TMS significantly increased activity in left V1 for invalidly cued right targets. Recall that the behavioural results presented previously (Fig. 2) had shown that perceptual discrimination of these same invalidly cued right targets was significantly enhanced during high vs. low TMS in the behavioural data. Now we observe that the same specific comparison (high vs. low right AG TMS, for right invalidly cued targets in particular) that led to enhanced visual performance behaviourally also led to enhanced BOLD in the target-responsive, contralateral left V1. This TMS-induced, condition-specific elevation of BOLD was also present in left V2, again for invalidly cued right targets; but did not reach significance for V3. We discuss the apparent tendency for stronger remote TMS effects upon earlier rather than higher visual areas later (see also Ruff *et al.*, 2006).

Thus, stimulation of the right AG increased the contralateral visual response in the early retinotopic cortex to a right target that had been preceded by an invalid (left) cue. As our analyses (and plots in Fig. 4) concern TMS-induced differences, this effect cannot reflect visual stimulation *per se*, because this was equivalent across the high- and low-intensity TMS conditions. Moreover, in accord with our behavioural TMS findings, there was no such effect for the otherwise analogous left targets, in the contralateral (right) visual cortex, when those left targets were invalidly cued (see leftmost three bars in Fig. 4).

These context-dependent TMS effects observed for invalidly cued targets were specific to the target-responsive retinotopic ROIs. No TMS-induced effects were observed in corresponding dorsal visual areas that represent the lower visual field instead (e.g. *P* > 0.3 for invalidly cued right targets in left dorsal V1), away from our upper-quadrant stimuli. Moreover, TMS-induced effects in the left ventral visual cortex were specific to invalidly cued right targets, being absent (e.g. *P* > 0.3 for left ventral V1) for validly cued right targets. Finally, no such effects were observed during the vertex-TMS experiment (e.g. *P* > 0.7 for invalidly cued right targets in left ventral V1).

## Discussion

We applied TMS over the right AG, or a control vertex site, during an event-related, exogenously cued visuospatial attention task. Rather than comparing TMS vs. none, we made the closer comparison of high- vs. low-intensity TMS at each site. Behaviourally, we found that (high- vs. low-intensity) TMS over the right AG selectively affected perceptual discrimination of right but not left visual targets, both outside and inside the scanner, in a manner that strongly depended on cue validity. Specifically, with the present TMS protocol in the present paradigm, high- vs. low-intensity TMS over the right AG boosted accuracy specifically for invalidly cued right targets, but not for validly cued targets (see Fig. 2). Thus, stimulation of the right AG evidently facilitated rightward spatial reorienting, for invalidly cued right targets. See also Hilgetag *et al.* (2001) and Chambers *et al.* (2006) for discussion of previous effects of parietal TMS on ipsilateral visual targets, and also for the more general point that TMS need not always lead to impairments of performance, but can also lead in some cases to specific improvements, as here.

By combining TMS with concurrent fMRI (Bohning *et al.*, 1999; Baudewig *et al.*, 2001; Ruff *et al.*, 2006, 2009; Sack *et al.*, 2007; Bestmann *et al.*, 2008a, b; Driver *et al.*, 2009), we were also able to examine the impact of our TMS manipulation on brain activity during task performance. This included any possible impact on areas remote from but interconnected to the right AG, and whether any such effects on brain activity might depend on the current attentional condition. With this approach, we found interhemispheric effects of the TMS (see also Bestmann *et al.*, 2008b; Blankenburg *et al.*, 2008) that depended strongly on the current attentional state. In particular, activity in the left AG (homologous to the targeted TMS site) was dependent on the interaction between TMS intensity, target hemifield and cue validity (see Fig. 3A), showing a similar three-way interaction as for the behavioural data. High-intensity TMS systematically increased the BOLD signal in the left AG for invalidly cued right targets (whereas the BOLD response here decreased instead for invalidly cued left targets). This pattern was specific to invalidly cued trials, did not occur when targets were validly cued, and survived cluster correction for the whole brain.

A similar pattern (also surviving cluster correction) was apparent in the posterior cingulate cortex and adjacent left precuneus, potentially indicating a role of these regions as cross-hemispheric integrative nodes. These structures are known to be extensively cross-connected to the parietal cortices in either hemisphere (Cavada & Goldman-Rakic, 1989; Morecraft *et al.*, 2004; Margulies *et al.*, 2009). They have also been implicated in the neglect syndrome (Mesulam, 1999), reflexive orienting (Dean *et al.*, 2004) and reorienting of attention or ‘circuit-breaking’ (Corbetta *et al.*, 2000; Corbetta & Shulman, 2002) in previous work.

The BOLD response of the visual cortex was studied in more detail via the individual retinotopic mapping of voxels responding to our target stimuli in mapped ventral V1–V3. This allowed us to determine whether right AG TMS might exert remote, condition-specific effects on the early visual cortex, and whether any such remote effects might be analogous to the pattern shown for visual performance. As for the remote BOLD effects in the left AG, target-responsive retinotopic ROIs in the left hemisphere (contralateral to right targets) were reliably modulated by TMS of the right AG, again in a manner that depended closely on the current attentional condition. Specifically, the BOLD response of left ventral V1 and V2 to invalidly cued right targets was enhanced during high-intensity TMS (see Fig. 4), in accordance with the improved performance for the same condition. Again, in line with the behavioural impact of TMS, no such effect was observed for left targets or on validly cued trials. Neither was the effect observed in dorsal visual areas (non-responsive to our upper-hemifield targets), being specific to the target-responsive retinotopic cortex instead. These remote TMS effects on the visual cortex suggest that the role of the right AG during reorienting of attention may involve modulation of stimulus responses in relatively early visual regions, possibly via the posterior cingulate cortex and left AG.

One potentially noteworthy aspect of the remote, condition-dependent TMS effects on the left ventral visual cortex (responding to right targets) was that the TMS effect appeared stronger (see Fig. 4, rightmost three bars) for the early visual cortex (V1) than for later areas (V3). This appears to be different from the standard findings of (TMS-unrelated) attentional modulation of visual responses that are typically stronger for later than earlier visual areas (cf. Kastner *et al.*, 1998). However, the present outcome appears potentially to be consistent with previous findings that some remote TMS effects can appear stronger for earlier than later areas in the visual cortex (e.g. Ruff *et al.*, 2006). Future extensions of the approach pioneered here could test more thoroughly how condition-specific remote TMS effects upon the visual cortex may vary for different levels of the cortical hierarchy, including use of more extensive mapping of visual areas than was possible here given the time constraints and scope of our study.

Taken together, our existing results already indicate a critical role for the right AG as one source of top-down visuospatial attention (see also Silvanto *et al.*, 2008), especially when reorienting of attention is required to the ipsilateral side. Our concurrent TMS–fMRI data revealed interhemispheric influences on the opposite parietal cortex, and modulation of target representations in the early visual cortex, which depended on the current event-related attentional condition. These data indicate that the behavioural consequences of parietal TMS may not depend solely on changes in local activity, but potentially also on modulation of remote interconnected brain regions (Ruff *et al.*, 2006, 2008, 2009; Sack *et al.*, 2007; Bestmann *et al.*, 2008a; Driver *et al.*, 2009). Here we showed that the remote interhemispheric consequences of right AG TMS alter in a dynamic fashion with the event-related state of attention.

In apparent contrast to the left AG, the BOLD signal in the stimulated right AG did not show a fully significant high-level three-way interaction between TMS intensity, target hemifield and cue validity, perhaps because local field distortions can potentially reduce the sensitivity of the fMRI signal immediately beneath the TMS coil (see Bestmann *et al.*, 2008a). Nevertheless, within a spherical ROI for the stimulated region of the right AG, we did observe an interaction of TMS intensity with cue validity, for right targets in particular, thus representing a somewhat weaker variant of the left AG pattern. Future research with increased power might be able to compare the left and right AG more directly.

It has long been suspected that interhemispheric interplay might be crucial for orienting of attention, based primarily on clinical inferences from brain-damaged patients hitherto (e.g. Kinsbourne, 1977). The present study produced a new type of evidence for the role of interhemispheric interplay in the normal brain, by uncovering left-hemisphere effects due to right AG TMS, which showed some analogy to performance effects of the same TMS for right targets contralateral to the left hemisphere. Critically, these effects were dynamic in that they depended on the current attentional state, excluding a simpler interhemispheric inhibition account (see also Kinsbourne, 1977). A potentially fruitful extension of our study would be to determine whether the left AG can have corresponding influences upon right-hemisphere structures, or whether instead the direction of causal influence in redirecting attention stems mainly from right-hemisphere influences upon the left hemisphere. Resolving this issue was beyond the scope of the present study, but might be addressed in future research now that we have provided a proof-of-principle for interhemisphere remote TMS effects, which depend on the current attentional condition. It may be that the right AG enjoys a privileged role in controlling reorienting of attention (see Corbetta *et al.*, 2000; Rushworth *et al.*, 2001; Macaluso *et al.*, 2002; Mort *et al.*, 2003a, b; Chambers *et al.*, 2004; Thiel *et al.*, 2004; Kincade *et al.*, 2005; Vuilleumier *et al.*, 2008), whereas the left AG, which we found to be influenced here, might be critical for responses to right targets in particular, if, as has been suggested, the left parietal hemisphere selectively represents contralateral space (Mesulam, 1999).

Regardless of such further considerations and possibilities for future experiments, the present data provides an existence-proof that the impact of TMS on behaviour and on remote brain areas (here in the opposite hemisphere to that stimulated) can depend critically and dynamically on event-related attentional conditions [cf. Blankenburg *et al.* (2010), who could only study blocked attentional conditions in their TMS–fMRI study]. Our findings illustrate that concurrent TMS–fMRI can provide a new approach to studying how causal interplay between brain regions may vary with the current cognitive state, in this case highlighting the role of interhemispheric influences for redirecting the spatial distribution of attention.

## Abbreviations

AG: angular gyrus
BOLD: blood-oxygen-level dependence
EPI: echo planar image
fMRI: functional magnetic resonance imaging
ROI: region of interest
TMS: transcranial magnetic stimulation

## Supporting Information

Fig. S1. TMS site specification in each participant.

Table S1. Main effects and interactions from the whole brain analysis.

Please note: As a service to our authors and readers, this journal provides supporting information supplied by the authors. Such materials are peer-reviewed and may be re-organized for online delivery, but are not copy-edited or typeset by Wiley-Blackwell. Technical support issues arising from supporting information (other than missing files) should be addressed to the authors.
