# Supplementary material for: Concurrent TMS–fMRI reveals dynamic interhemispheric influences of the right parietal cortex during exogenously cued visuospatial attention
Source: Eur J Neurosci. 2011 Mar;33(5):991–1000. doi: 10.1111/j.1460-9568.2010.07580.x (PMC3437477; doi:10.1111/j.1460-9568.2010.07580.x)
Supplement: Supplementary file 1 [file ejn0033-0991-SD1.doc]

# SUPPLEMENTARY RESULTS

**<Insert Figure S1 here>**

**Figure S1. TMS site specification in each participant.**

Stimulation sites for TMS shown on the rendered cortical surface of each individual participant in native space. The right AG site is shown in red, the vertex control site in blue. The neighbouring intra parietal sulcus (IPS) is marked in pink. The first five participants (P1-P5, as labeled with green characters) are those who also participated in the concurrent TMS-fMRI experiment and the retinotopic mapping.

| **Table S1**  **Summary of peak coordinates and p-values for major contrasts from the whole brain analysis** | | | | | | |
| --- | --- | --- | --- | --- | --- | --- |
| Anatomical/functional region | MNI coordinates | | | Peak voxel | | Cluster |
|  | x | y | z | T score | P value (uncorrected at voxel level) | **P value**  **(corrected on cluster level)** |
|  | | | | | | |
| Left minus right hemifield targets  R Occipital Cortex    R Inferior Parietal Lobe | 20  28  52  38 | -66  -72  -72  -48 | -10  -13  3  55 | 10.46  8.74  7.13  4.12 | < .001  < .002 | *<. 001 |
| Right minus left hemifield targets  L Occipital Cortex | -26  -24  -48 | -70  -84  -72 | -13  25  10 | 12.15  8.20  6.54 | < .001 | *< .001 |
| TMS high minus low intensity  R superior temporal gyrus  L superior temporal gyrus | 62  -64 | -14  -22 | 8  15 | 4.12  4.02 | < .001  < .001 |  |
| 3-way interaction (TMS intensity x cue validity x target hemifield, see main text)    L Angular Gyrus      BL Posterior Cingulate Corte  L Precuneus  R Superior Temporal Sulcus    L Occipital Cortex | -36  -36  -44  2  -8  56  -10 | -76  -84  -76  -60  -74  -70  -68 | 50  40  35  25  33  25  0 | 5.06  4.54  3.71  4.93  3.94  4.31  3.59 | < .001  < .001  < .001  < .001 | *< .002  *< .001 |
| *** significant when whole-brain corrected on cluster level at p< .05 or lower**, i.e. for the threshold used in the main text and the whole-brain figures | | | | | | |

**Table S1: Main effects and three-way interaction for TMS over right AG but not for vertex, from the whole-brain analysis**

The main effect of target hemifield (left or right) revealed BOLD response in left occipital cortex for the right minus left contrast, as expected, including lingual and fusiform gyri, extending dorsally into the middle and superior occipital gyri (*p*< .001 corrected on cluster level). Similar areas were activated in right occipital cortex for the reverse contrast, with activation extending to inferior parietal areas. As also expected from previous findings, the main effect of TMS intensity (low or high) revealed activation of bilateral auditory cortex. This has been reported in previous concurrent TMS-fMRI studies due to the ‘click’ sound associated with TMS discharge, which is inevitably slightly louder in the high TMS condition (Bestmann et al., 2008; Ruff et al., 2006; Baudewig et al., 2001; Bohning et al., 2000). The activations observed in left AG and bilateral Posterior Cingulate Cortex for the critical three-way interaction (TMS intensity × target hemifield x validity, analogous to the behavioural pattern, see main text) were specific for right AG TMS, as they were not observed during TMS of the control vertex site (all p> .4). Smaller activation clusters were observed in right STS and left calcarine cortex at lower uncorrected threshold after the right AG TMS, but not for vertex TMS. See main text.

Reference List

Baudewig J, Siebner HR, Bestmann S, Tergau F, Tings T, Paulus W, Frahm J (2001) Functional MRI of cortical activations induced by transcranial magnetic stimulation (TMS). Neuroreport 12:3543-3548.

Bestmann S, Swayne O, Blankenburg F, Ruff CC, Haggard P, Weiskopf N, Josephs O, Driver J, Rothwell JC, Ward NS (2008) Dorsal premotor cortex exerts state-dependent causal influences on activity in contralateral primary motor and dorsal premotor cortex. Cereb Cortex 18:1281-1291.

Bohning DE, Shastri A, Wassermann EM, Ziemann U, Lorberbaum JP, Nahas Z, Lomarev MP, George MS (2000) BOLD-f MRI response to single-pulse transcranial magnetic stimulation (TMS). J Magn Reson Imaging 11:569-574.

Ruff CC, Blankenburg F, Bjoertomt O, Bestmann S, Freeman E, Haynes JD, Rees G, Josephs O, Deichmann R, Driver J (2006) Concurrent TMS-fMRI and psychophysics reveal frontal influences on human retinotopic visual cortex. Curr Biol 16:1479-1488.
