# Supplementary figures and images for: Concurrent TMS–fMRI reveals dynamic interhemispheric influences of the right parietal cortex during exogenously cued visuospatial attention
Source: Eur J Neurosci. 2011 Mar;33(5):991–1000. doi: 10.1111/j.1460-9568.2010.07580.x (PMC3437477; doi:10.1111/j.1460-9568.2010.07580.x)

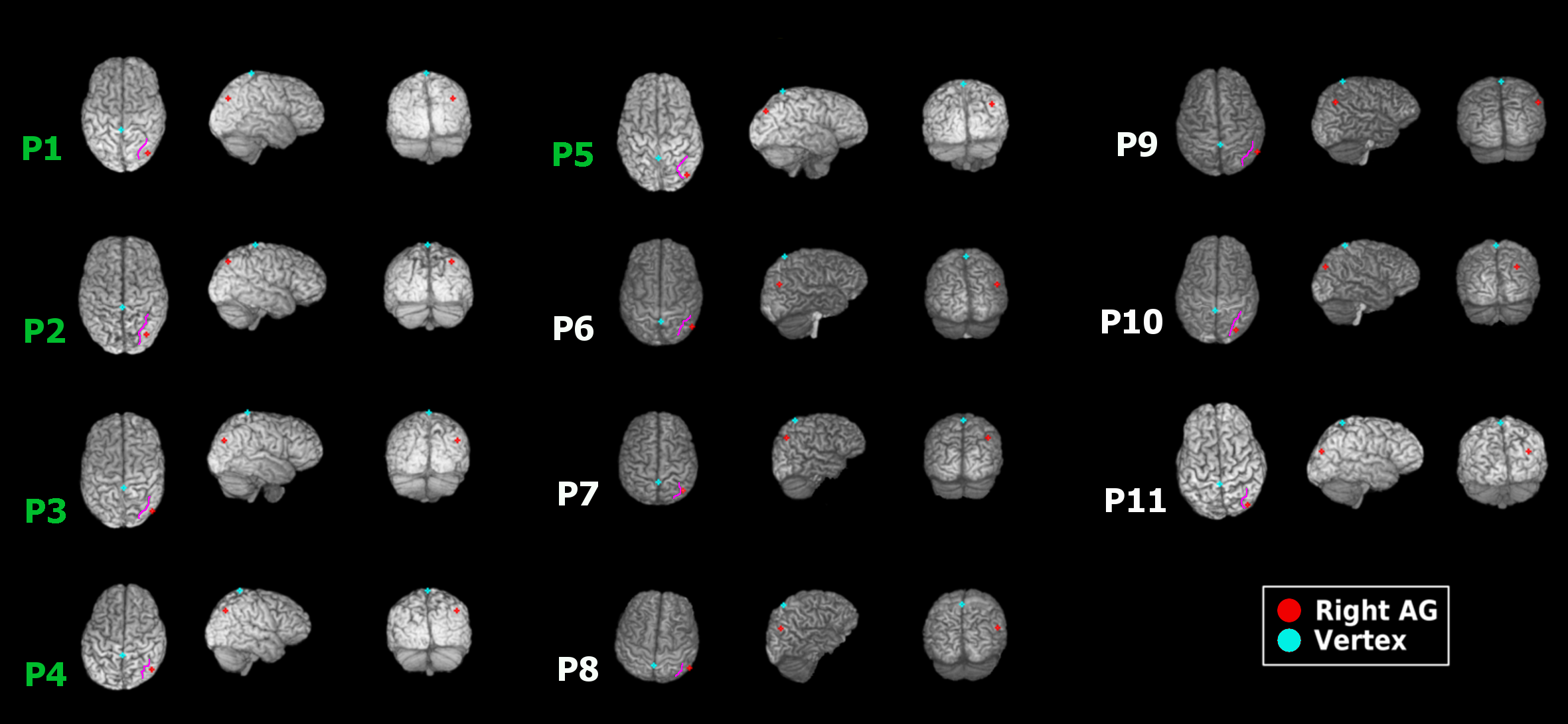

Supplement: Supplementary file 2 [file ejn0033-0991-SD2.tif]
